# Parvimonas micra Bacteremia in the Setting of a Hepatic Abscess: A Case Report

**DOI:** 10.7759/cureus.56497

**Published:** 2024-03-19

**Authors:** Aneil S Walizada, Sarah E Lyons, Chulou Penales, Carlos Lopez

**Affiliations:** 1 Internal Medicine, Hospital Corporation of America (HCA) Healthcare Westside Regional Medical Center, Plantation, USA; 2 Internal Medicine, Nova Southeastern University Dr. Kiran C. Patel College of Osteopathic Medicine, Davie, USA

**Keywords:** antibiotics, sepsis, hepatic abscess, bacteremia, parvimonas micra

## Abstract

*Parvimonas micra* is a Gram-positive anaerobic coccus that typically colonizes the oral cavity and gastrointestinal tract in humans. Though *P. micra *is typically associated with periodontal abscesses, it can also be an unlikely cause of bacteremia. Here, we report a case of *P. micra *bacteremia in the setting of a hepatic abscess. Antibiotic treatment of the bacteremia was initiated, and the entry source of the infection was investigated using various imaging techniques in the inpatient setting. A hepatic abscess was suspected to be the origin of infection for the *P. micra *bacteremia. Successful antibiotic treatment was confirmed by negative repeat blood cultures and an improvement in the patient’s symptoms and clinical picture.

## Introduction

*Parvimonas micra* is a Gram-positive anaerobic coccus that frequently colonizes the oral cavity and gastrointestinal tract in humans. The treatment and origin of infection in cases involving *P. micra* are scarcely researched. Reports of bacteremia in the setting of this microbe are uncommon. Few reports, however, have been published establishing an association between *P. micra* bacteremia and colonic carcinoma, pneumonia, empyema, and osteomyelitis [1-5]. This case report details the clinical course and management of a patient who had *P. micra* bacteremia in the setting of a hepatic abscess.

## Case presentation

An elderly male with a medical history significant for coronary artery disease with stent placement, congestive heart failure with reduced ejection fraction, atrial fibrillation, myocardial infarction, hyperlipidemia, hypertension, and chronic neck and back pain secondary to cervical and lumbar stenosis initially presented to the hospital with complaints of fever, chills, anorexia, generalized weakness, nausea, vomiting, shortness of breath with cyanosis, and flank pain of three days duration. The patient denied chest pain, palpitations, or any other symptoms at the time of presentation. On arrival at the emergency department (ED), emergency medical services reported the patient’s oxygen saturation to be in the 80’s while being transported, which improved after treatment with a non-rebreather mask. The patient reported being a former smoker but denied any alcohol or recreational drug use. The patient’s surgical history was significant for cardiac catheterization with seven cardiac stents, automatic implantable cardioverter-defibrillator (AICD) placement, kidney stenting, and laminectomy. The patient denied any recent dental procedures. His chronic medical conditions were managed using his home outpatient medications.

In the ED, the patient's vitals showed a temperature of 37.7 degrees Celsius (99.86 degrees Fahrenheit), a pulse of 81 beats per minute, a respiratory rate of 18 respirations per minute, and a blood pressure of 153/66 mmHg. The patient was admitted for further medical management. Extensive imaging was ordered to rule out acute pathologies related to the patient’s clinical presentation. The patient’s labs were monitored throughout his hospital stay, as listed in Table 1.

**Table 1:** The patient's lab values throughout his hospital stay * Indicates lab value outside of set reference range per hospital standards; - Indicates lab was not drawn that day WBC: white blood cells; BUN: blood urine nitrogen; AST: aspartate transaminase; ALT: alanine transaminase; ALP: alkaline phosphatase

	Day 1	Day 2	Day 3	Day 4	Day 5	Day 6	Day 7	Reference range
WBC (10^3 uL)	8.8	*12.8	*11.1	*10.9	*12.1	*15.2	*10.9	4.0-10.5
Hemoglobin (g/dL)	*10.8	*10.2	*10.0	*10.3	*10.8	*10.5	*11.0	13.7-17.5
Sodium (mmol/L)	137	136	138	137	136	138	137	135-145
Potassium (mmol/L)	3.9	4.1	3.8	4.4	4.1	4.3	4.5	3.5-5.2
BUN (mg/dL)	*28	*42	*38	*31	*32	*37	*35	22-Jun
Creatinine (mg/dL)	*1.23	*1.7	*1.34	0.9	1	1.06	1.1	0.43-1.13
Lactic acid (mmol/L)	*2.1	-	-	1.7	-	-	-	0.4-2.0
Magnesium (mg/dL)	1.8	-	-	-	-	-	-	1.6-2.4
Total bilirubin (mg/dL)	1.1	0.5	-	0.6	0.5	0.4	0.6	0.1-1.2
AST (units/L)	*67	*62	-	*60	*127	*52	*114	Oct-40
ALT (units/L)	51	55	-	58	*66	57	50	Oct-60
ALP (units/L)	*164	103	-	113	*134	104	119	20-130
Albumin (g/dL)	3.7	2.8	-	*2.4	*2.8	*2.7	*2.7	3.2-5.0
Lipase (U/L)	44	-	-	-	-	-	-	23-300

The patient’s electrocardiogram indicated a ventricular-paced rhythm. The chest X-ray exhibited cardiomegaly with pulmonary vascular congestion and bibasilar interstitial opacities, which was suspected at the time to be on the basis of pulmonary edema or atypical pneumonia. A computerized tomography (CT) scan of the chest revealed a single right-sided pulmonary nodule and mild areas of atelectasis in the lower lobes. A CT scan of the abdomen and pelvis revealed a 1.7 cm low attenuation lesion in the pancreatic neck, which was suspected at the time to represent a side-branch intraductal papillary mucinous neoplasm (IPNM) or peripancreatic lymph node. The CT scan of the abdomen and pelvis also showed a Spigelian hernia containing portions of the sigmoid colon, without evidence of obstruction, as well as diverticulosis without evidence of acute diverticulitis. The CT lumbar spine showed chronic degenerative changes without evidence of acute fractures or complications. A nasal cannula, non-rebreather mask, and bilevel-positive airway pressure (BiPAP) treatments were used at separate times throughout the patient’s hospital stay to maintain oxygen saturation above 92%. 

**Figure 1:**
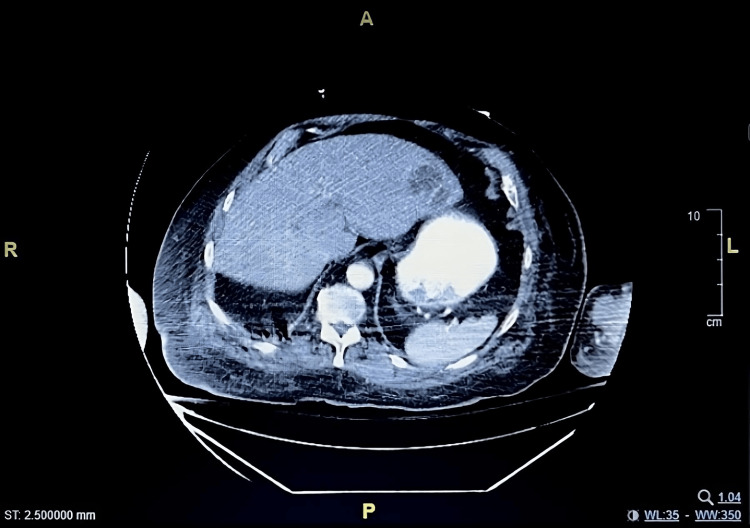
Contrast-enhanced computed tomography in axial view shows a multi-septated cystic structure in the left lobe of the liver measuring 4.5 x 2.7 cm.

One day following admission, the patient developed leukocytosis, which was coupled with lactic acidosis, elevated creatinine, and elevated aspartate aminotransferase (AST), raising concern for severe sepsis. Additionally, two sets of the patient’s blood cultures were positive for *Parvimonas micra*. Given these findings, treatment was initiated for severe sepsis in the setting of *P. micra* bacteremia. The patient was empirically started on cefepime and azithromycin in the ED but was switched to piperacillin/tazobactam (Zosyn) following positive blood cultures. To investigate the source of infection, further imaging was obtained, including a transesophageal echocardiogram (TEE), which ruled out endocarditis. A CT scan of the abdomen and pelvis with contrast revealed a multi-septated cystic structure in the left lobe of the liver concerning for a hepatic abscess, measuring 4.5 x 2.7 cm (Figure 1). The cyst was identified as the likely source of the bacteremia. An ultrasound-guided percutaneous left hepatic drain was placed as a therapeutic and diagnostic measure to treat the hepatic abscess. The purulent fluid obtained from the abscess was negative for any organisms; however, antibiotic treatment was initiated five days prior to the abscess drainage. Another potential source of the *P. micra* bacteremia could have been the atypical pneumonia indicated by the patient’s initial chest X-ray. 

## Discussion

Current literature identifies the oral and gastrointestinal tract as common sources of infection for *P. micra* bacteremia in humans. Intra-abdominal sources have also been identified as a cause of this bacteremia [6]. Many prior case reports regarding *P. micra* bacteremia were in the setting of immunocompromised patients or in patients with numerous comorbidities [1, 2, 5]. Similarly, the patient in this report had several comorbidities that made him vulnerable to this opportunistic pathogen, including his advanced age and extensive history of cardiac disease. Bacteremia caused by this pathogen is typically not fatal if antibiotic treatment and abscess drainage, if applicable, are initiated in a timely manner. The patient in this report was treated with Zosyn (piperacillin/tazobactam) in an inpatient setting and was discharged home with IV antibiotics. The improvement in the patient’s symptoms and clinical picture, coupled with the successful hepatic abscess drainage and negative repeat blood cultures towards the end of the patient’s hospital stay, allowed the patient to be medically optimized for discharge home.

## Conclusions

In conclusion, prompt diagnosis and tailored antimicrobial therapy are essential for a positive clinical outcome in patients diagnosed with *P. micra* bacteremia. Investigation of the origin of infection is also warranted, as studies have established an association between *P. micra* bacteremia and several different organ systems as the infection origin. *Parvimonas micra* is typically susceptible to treatment with antimicrobial agents, but antibiotic selection should be based on sensitivity testing to ensure adequate bacterial coverage. In the future, for patients presenting similar to the case detailed in this report, it is imperative to have a high suspicion for *P. micra* bacteremia to ensure early imaging, diagnosis, and treatment of this potentially fatal infection.

## References

[1] Khan MS, Ishaq M, Hinson M, Potugari B, Rehman AU (2019). Parvimonas micra bacteremia in a patient with colonic carcinoma. Caspian J Intern Med.

[2] Yu Q, Sun L, Xu Z, Fan L, Du Y (2021). Severe pneumonia caused by Parvimonas micra: a case report. BMC Infect Dis.

[3] Feng Y, Wu C, Huang X, Huang X, Peng L, Guo R (2022). Case report: successful management of Parvimonas micra pneumonia mimicking hematogenous Staphylococcus aureus pneumonia. Front Med (Lausanne).

[4] Yamada K, Taniguchi J, Kubota N (2023). Empyema and bacteremia caused by Parvimonas micra: a case report. Respir Med Case Rep.

[5] Itoh N, Akazawa N, Ishibana Y, Hamada S, Hagiwara S, Murakami H (2022). Femoral osteomyelitis caused by oral anaerobic bacteria with mixed bacteremia of Campylobacter rectus and Parvimonas micra in a chronic periodontitis patient: a case report. BMC Infect Dis.

[6] Watanabe T, Hara Y, Yoshimi Y, Fujita Y, Yokoe M, Noguchi Y (2020). Clinical characteristics of bloodstream infection by Parvimonas micra: retrospective case series and literature review. BMC Infect Dis.

